# Food selectivity in Autism Spectrum Disorder: implications of eating, sensory and behavioural profile

**DOI:** 10.3389/fpsyt.2025.1587454

**Published:** 2025-06-02

**Authors:** Maria Pia Riccio, Maria Marino, Raffaele Garotti, Annalisa Tassiello, Valeria Maffettone, Mariangela Pezone, Carmela Bravaccio

**Affiliations:** ^1^ Department of Maternal and Child Health, Child Neuropsichiatry, Azienda Ospedaliera Universitaria (AOU) Federico II, Naples, Italy; ^2^ Cognitive Psychotherapy School, Scuola di Psicoterapia Cognitiva (SPC), Naples, Italy; ^3^ Department of Mental and Physical Health and Preventive Medicine, University of Campania “Luigi Vanvitelli”, Naples, Italy; ^4^ Department of Medical and Translational Sciences, Child Neuropsychiatry, Federico II University, Naples, Italy; ^5^ Department of Psychology, University of Campania “Luigi Vanvitelli”, Naples, Italy

**Keywords:** Autism Spectrum Disorder, clinical phenotype, food selectivity, sensory profile, challenging behavior, intellective quotient

## Abstract

**Introduction:**

To characterise possible clinical associations between food selectivity, a typical aspect of Autism Spectrum Disorder (ASD), and certain commonly observed aspects of the phenotype: sensory abnormalities, challenging behaviour, autistic symptom intensity and Intellective Quotient.

**Methods:**

The present is a retrospective observational study. Fifty-two ASD subjects were enrolled that underwent a comprehensive psychodiagnostic assessment including the *Short Sensory Profile* questionnaires (to assess sensory abnormalities), the *Aberrant Behaviour Checklist* (to assess challenging behaviour) and the *Food Preference Inventory* (to assess food selectivity). The possible association between food selectivity, the intensity of autistic symptoms and the presence or absence of associated Intellective Disability was also subsequently assessed. Any differences between female and male were also assessed.

**Results:**

Statistically significant correlations were observed between tactile, gustatory and olfactory sensitivity and food selectivity. These modalities were also found to predict greater food selectivity on regression analysis. No significant correlations were observed between the behavioural profile, intellective quotient and intensity of autistic symptomatology with food selectivity. In addition no significant scores were observed on the basis of sex.

**Discussion:**

As far as can be seen from the results, the food selectivity of ASD subjects appears to be underlain mainly by sensory abnormalities and does not correlate with other clinical aspects of ASD subjects. Despite this, a comprehensive assessment of the various phenotypical aspects is still of fundamental importance. Further studies that could lead to a progressive characterisation of the phenotypical aspects of ASD subjects in order to provide increasingly personalised treatment are therefore considered useful.

## 1 Introduction

Autism Spectrum Disorder (ASD) is a neurodevelopmental disorder with persistent deficits in communication and social interaction, patterns of behavior, interests or activities that are restricted and/or repetitive. These symptoms must also be present in the early period of development and must result in clinically significant impairment of the individual’s functioning in various life contexts (1). Individuals with ASD may present a number of psychiatric comorbidities, among which the most frequent are Attention Deficit/Hyperactivity Disorder (ADHD) and mood disorders and anxiety disorders (2). Medical comorbidities such as epilepsy, asthma, atopy and gastrointestinal diseases may also be present, with an increased incidence compared to the general population (3). Among the characteristics associated with ASD, Food Selectivity (FS) is one of the major clinical features, with an estimated prevalence between 51% and 89% in autistic children (4), with an incidence that is significantly higher in individuals with ASD than in children with a neurotypical development (5). According to this, in the clinical assessment of these patients, it is essential to evaluate this aspect since it is related with nutritional deficiencies (e.g. Vitamin D, Vitamin E, micronutrients) (6). The range of dysfunctional eating behaviours presented by subjects ASD relates not only with FS, but also with obsessive behaviors, such as adherence to rigid meal routines, dysreactive behaviors at the time of offering food and pica (4). In addition, there is evidence that abnormal feeding relates with gastrointestinal problems, typical of patients ASD, such as gastroesophageal reflux, abdominal pain, constipation (7). According to *Bandini* et al. (2010) FS includes three separate categories with different characteristics: food refusal; limited food repertoire; high-frequency single food intake. The last expression refers to the introduction of the same food at least 4–5 times a day (8) and it’s the most represented eating behavior in children ASD. Moreover, food refusal could be related to the characteristics of the food, such as its texture, temperature, color, shape, smell or taste (9). Therefore, this aspect relates with one of the diagnostic criteria of ASD according to the DSM-5, since in the B criteria it’s included an *“hyper- or hypo-responsiveness in response to sensory stimuli or unusual interests toward sensory aspects of the environment”* (1). It has been shown that abnormalities of the sensory profile, most of all of the sense of taste, lead to greater food refusal and lower consumption of certain food, such as vegetables (10). According to some authors, sensory abnormalities are also represented in 90% of the patients ASD (11) and are linked to both FSy and dysfunctional behaviours (12). Given these premises, we hypothesised that subjects with greater FSy may exhibit not only higher intensity sensory abnormalities, but also a worse behavioral profile. There is evidence of this relation in literature, though sensory and behavioral profiles have never been evaluated together but in disjunct form (13–15). Secondly, we hypothesised that FS may be a typical characteristic of individuals with ASD, that could be directly associated with autistic symptomatology. Finally, we hypothesised that FS might vary based on sex and cognitive level.

## 2 Patients and methods

### 2.1 Subjects

This is a retrospective observational clinical study. We enrolled subjects referring to the Childhood and Adolescent Neuropsychiatry Unit of the University of Naples ‘Federico II’. The subjects were selected from patients consecutively referred to the specialist outpatient clinic dedicated to ASD for psychodiagnostic assessments or clinical monitoring between January 2022 to January 2023. Inclusion criteria were: diagnosis of ASD in accordance with DSM-5; age between 4 and 15 years old; stable rehabilitation treatment for at least 6 months at the moment of the visit; assessment using standardised tests (including nutritional, behavioural and sensory evaluation); written informed consent from caregivers. Exclusion criteria were: comorbidity for neurological disorders; comorbidity with Attention Deficit/Hyperactivity Disorder (ADHD) or other developmental psychiatric disorders; presence of swallowing difficulties; presence of gastrointestinal diseases that could affect feeding (p.e. celiac disease); ongoing psychopharmacological treatment for psychiatric or behavioral comorbidities; individuals on a special diet (e.g. casein-free diet, gluten-free diet, etc.); caregivers refusing to sign informed consent. Among the exclusion criteria we added the presence of comorbidity with ADHD in order not to have bias in the assessment of the behavioral profile (see *section 2.3*). We did not take into account comorbidity with Intellective Disability (ID) or Language Disorders because, according to DSM-5, these two represent specifiers to be added to the diagnosis of ASD. 120 subjects with a diagnosis of ASD were admitted between January 2022 to January 2023. 68/120 subjects were excluded because they did not meet the inclusion and exclusion criteria established *a priori* for the study. Final sample consisted of 52 subjects. Data collected concerned results of clinical evaluation carried out by: Autism Diagnostic Observation Schedule-2 (ADOS-2) test (16) and Social Responsiveness Scale (SRS) questionnaire (17) aimed at detecting and classifying the presence of autistic symptoms; development/intellective tests such as Griffiths Mental Development Scale III edition (18), Leiter International Performance Test- Revised (Leiter-R) (19) and Wechsler Intelligence Scale for Children - Fourth Edition (WISC-IV) (20); Vineland Adaptive Behavior Scales-II edition (VABS-II) (21), to evaluate adaptive competence. These tools are routinely used for subjects coming to our facility for diagnostic evaluation. Patients were also categorised according to the DSM specifiers, distinguishing the presence or absence of intellective and language impairment. The study was conducted in according to principles of Helsinki Declaration; ethical approval was obtained by the Ethics Committee of the University Federico II of Naples (research protocol No.78/2024). Participation in the study was free and unconditioned by the investigators. Data were collected and analysed in an anonymous way. Written informed consent was collected from parents or caregivers of enrolled children for both clinical information collection and data acquisition and treatment.

### 2.2 Nutritional assessment

In order to assess the presence or absence of FS in our sample, we collected Food Preference Inventory (FPI) results. FPI is a caregiver report checklist of preferred or typically accepted food items from each of the five food groups (fruits, vegetables, dairy, proteins, carbohydrates, and miscellaneous mixed food items -e.g. stew) (22). This scale allows parents to indicate the frequency with which a particular food is consumed (daily, weekly, monthly, not consumed); there is also an additional scale that allows them to indicate whether that food is consumed within the family or not, in order to discriminate whether a food is not consumed due to non-proposition or refusal. As previously described by *Riccio M.P.* et al. (2018), we used the cut off of 46.5 in order to discriminate subjects with FS from those who did not present this characteristic (23).

### 2.3 Behavioural assessment

The enrolled subjects were assessed through the Aberrant Behavior Checklist (ABC) (24) in order to evaluate the behavioral profile. This is a 58-item checklist subdivided into 5 subscales (Irritability; Lethargy/Social withdrawal; Stereotyped behaviors; Hyperactivity, lack of compliance; Inappropriate language) developed to evaluate the response to specific treatments or to study in general dysfunctional behaviors in subjects with developmental disorders. Each item is assigned a progressive number from 0 to 3 in order to indicate the severity of the required behavior in terms of intensity and frequency. In the comparative analysis, the mean scores of each subscale and the total score were used in order to evaluate statistically significant differences. This scale has also been validated for use in individuals with ASD (25).

### 2.4 Sensory profile assessment

We collected data of sensory profile, evaluated by the questionnaire Short Sensory Profile (SSP) (26): this is a short form of the “Sensory profile” questionnaire devised by Dunn (27), developed as a screening tool for sensory abnormalities of subjects up to 14 years of age. This tool consists of 38 items divided into 7 subscales (“Tactile sensitivity,” “Taste/Olfactory sensitivity,” “Motion sensitivity,” “Hyporesponsiveness/Sensation seeking,” “Auditory filtering,” “Low energy/Weakness,” “Visual/Auditory sensitivity”) and a final scale calculated from the sum of the individual values. Each item is assigned a progressive number from 1 to 5 according to frequency. In accordance with the standardisation of the questionnaire, it is possible to divide the single subscales and the final score into three categories: “Typical performance”, “Probable difference” and “Certain difference”, in which the difference found represents an altered or a probably altered sensory profile.

### 2.5 Statistical analysis

Descriptive statistics (frequencies, percentages, mean and standard deviations) were used to describe the sample. We performed the Shapiro-Wilks test for all variables under study, in order to assess whether the distribution was normal or not. Based on the distribution found, bivariate correlation analyses were set up for the variables of the SSP (including individual subscales), the ABC (including individual subscales) and the total score for FPI. Specific functions were set after evaluation of the variance distribution (specifically we used Pearson’s function for normally distributed values and Spearman’s Rho function for non-normally distributed values). For all statistically significant values, eventual regression analysis was also subsequently performed in order to assess the predictor role of the variables. For all set analyses, the goodness-of-fit of the constructed predictive model was also evaluated by assessing: 1) whether the residuals had normal distribution; 2) non-correlation of the independent variables to the error; 3) homogeneity of the variance of the residuals; 4) the linear distribution of the residuals; 5) presence of outliers that would invalidate the model (by Cock Distance Value); and 6) lack of autocorrelation of the residuals (by Durbin-Watson value). In order to analyse possible associations with the intensity of autistic symptomatology, bivariate analyses were also performed between FPI values and the variables: 1) comparison score taken on the ADOS-2 test; 2) scores taken on the SRS questionnaire (both total score and individual subscales). Finally, the distribution of the FPI score was also evaluated to assess some statistically significant differences within the evaluation group. Specifically, it was analysed whether: 1) subjects ASD could present different FPI scores based on gender (thus dividing the sample into male/female); 2) subjects ASD could present different FPI scores based on the presence/absence of concomitant ID. The presence of ID was determined based on the cognitive assessment performed and defined as IQ score < 70. For subjects to whom such an assessment could not be done (because, for example, they were particularly impaired or uncooperative), the cognitive level was clinically assessed on the basis of developmental and adaptive skills achieved. Results with p-value <0.05 were considered statistically significant in the comparison analysis. We applied The Statistical Package for Social Sciences Software 26th edition (SPSS-26th ed.) to perform statistical analysis.

## 3 Results

### 3.1 Result of descriptive analysis

Our sample consisted of 41 male subjects (79%). The mean age was 6.97 ± 2.45 years.The distribution according to levels of severity appeared homogeneous in the sample, as did the impairment of language. However, a higher prevalence of subjects without associated ID was observed (n=38, 73.1%) than subjects with associated ID. With reference to FS, this was assessed, as mentioned above, with the FPI questionnaire. In our sample, 25 subjects (48%) didn’t reach the cut-off, while for 27 subjects (52%) we found presence of FS. The clinical features of the sample are summarised in **Table 1**.

**Table 1:** Clinical features of ASD children.

	*ASD (n=52)*
Male Female	41 (79)^1^ 11 (21)^1^
Age, Months	86,63 (29,41)^2^
Autism Severity Index (According to DSM-5 severity levels) *Level 1* *Level 2* *Level* 3	20 (38,5)^1^ 23 (44,2)^1^ 9 (17,3)^1^
Children with verbal Impairment	28 (53,8)^1^
Children without verbal impairment	24 (46.2)^1^
Children with intellective/development impairment	14 (26.9)^1^
Children without intellective/development impairment	38 (73,1)^1^

^1^Expressed as number of subjects and percentage in parenthesis; ^2^Expressed as Mean and Standard Deviation in parenthesis.

### 3.2 Evaluation of the distribution of variables

Based on the Shapiro-Wilks test, we observed that the variables SSP, ABC and SRS (both total score and individual subscales) and the ADOS-2 comparison score had a non-normal distribution (p-value <0.05). Therefore, to carry out the correlation analysis, Spearman’s functions were chosen for the correlations between FPI and the variables SSP, ABC, SRS, ADOS-2 comparison score. Finally, in the comparison analysis of FPI for presence/absence of ID and sex, the t-test function for independent samples was used.

### 3.3 Results of the bivariate correlation analysis between the values of FPI, SSP and ABC

Spearman’s correlation analysis carried out between the values obtained at the SSP (including individual subscales) and the FPI revealed statistically significant results in the “Tactile Sensitivity” “Gustatory/Olfactory Sensitivity” “Hyporesponsiveness/Sensation Seeking” “Auditory Filtering” “Low Energy/Weakness” and the “Total Score” scales. The results are summarised in **Table 2**. Linear regression analysis was also set for all statistically significant correlations. Given what has already been described in the literature (9), sensory abnormalities could be one of the causes of FS in ASD subjects. Therefore, linear regression was performed by setting the FPI score as the dependent variable and the scores obtained at the SSP as independent (or predictor) variables. In the regression model used, the role of all independent variables was assessed separately. All variables that were already significant in the correlation analysis were also significant in the regression analysis (p-value < 0.05). The results of the linear regression analysis are shown in **Table 3**. Based on the assumptions given for linear regression (see 2.5 section), we had to reject the observed significance for the “Auditory Filtering” and “Low Energy/Weakness” scales due to lack of homogeneity of the variance of the residuals for both scales. All other scales met the criteria listed in 2.5 section. Therefore, the observed model was considered reliable. Since what was observed for the variable “Hyporesponsiveness/sensory seeking” was considered an unexpected result, further assessments were made to evaluate possible confounding factors. Specifically, after evaluation of the items asked in the questionnaire, it was found that this scale mainly refers to the behavioral connotation associated with sensory anomalies. New bivariate correlation according to Spearman was therefore carried out, by evaluating the possible correlation between IQ and this scale, observing a statistically significant direct correlation (Rho: 0.267, p-value: 0.043). In addition, in order to assess any confounding role of IQ on the observed outcome, a new regression analysis was performed between the scores of the “Hyporesponsiveness/Sensation Seeking” scale and the FPI, this time weighing the analysis on the IQ values associated with the individual subjects. This analysis found no statistical significance: thus, it is concluded that what has been observed above is due to a confounding effect of cognitive level. As further evidence of this, regression analysis, weighted on IQ scores, was also performed for the “Gustatory/Olfactory Sensitivity” “Tactile Sensitivity” and “Total” scales without resulting in any change in the statistical significance already observed. The results of the weighted linear regression analysis on the IQ are summarised in **Table 4**. From the bivariate correlation analysis. according to Spearman, conducted between the ABC variables (total score and individual subscales) and FPI values, no statistically significant results were observed (p-value > 0.05). Therefore, no further regression analysis was conducted. The results of this analysis are set out in **Table 5**.

**Table 2:** Results of bivariate correlation analysis between the values obtained at the SSP and FPI questionnaires; results are presented as rho (p-value).

	Tactile Sensitivity	Gustatory/olfactory sensitivity	Motion Sensitivity	Hyporesponsiveness/Sensation seeking	Auditory Filtering	Low energy/weakness	Auditory Sensitivity	Total
FPI	0.312 (0.016)*	0.594 (<0.01)*	0.191 (0.148)	0.310 (0.017)*	0.299 (0.021)*	0.263 (0.045)*	0.228 (0.082)	0.491 (0.01)*

*Statistically significant value (p-value <0.05). FPI, Food Preference Inventory; SSP, Short Sensoy Profile.

**Table 3:** Results of linear regression analysis; SSP scales were used as predictors; the FPI scale was used as the dependent variable.

Short Sensory Profile's Subscales	R2	B (CI)	p
Tactile Sensitivity	0.095*	1.39 (0.25-2.53)	0.018
Gustatory/olfactory sensitivity	0.372*	2.59 (1.69-3.49)	<0.01
Hyporesponsiveness/Sensation seeking	0.059*	0.957 (0.06-1.85)	0.036
Auditory Filtering	0.097*	1.42 (0.27-2.56)	0.016
Low energy/weakness	0.069*	0.83 (0.02-1.64)	0.044
Total	0.239*	0.432 (0.23-0.64)	0.01

*Statistically significant values (p value <0.05). CI, Confidence Interval; SSP, Short Sensoy Profile; FPI, Food Preference Inventory.

**Table 4:** Results of linear regression analysis; SSP scales were used as predictors; the FPI scale was used as the dependent variable.

Short Sensory Profile's Subscales	R2	B (CI)	p
Tactile Sensitivity	0.25*	2.26 (0.73-3.75)	0.005
Gustatory/olfactory sensitivity	0.32*	2.34 (1.02-3.66)	0.001
Hyporesponsiveness/Sensation seeking	0.081	0.95 (-0.09-1.99)	0.075
Total	0.35*	0.548 (0.26-0.83)	<0.01

All regression analyses were weighted on the IQ. *Statistically significant values (p value <0.05). CI, Confidence Interval; SSP, Short Sensory Profile; FPI, Food Preference Inventory.

**Table 5:** Results of bivariate correlation analysis between the ABC scales and FPI questionnaires; results are presented as rho (p-value).

	Irritability, agitation, crying	Social withdrawal, lethargy	Stereotyped behaviour	Hyperactivity/lack of compliance	Inappropriate language	Total
FPI	-0.116 (0.396)	-0.133 (0.330)	-0.022 (0.874)	-0.102 (0.452)	-0.133 (0.328)	-0.133 (0.329)

*Statistically significant values (p value <0.05). FPI, Food Preference Inventory; ABC, Aberrant Behavior Checklist.

### 3.4 Results of bivariate correlation analysis between FPI and autistic symptom intensity

Bivariate correlation analysis according to Spearman revealed no statistically significant values for either the SRS variables (Social Awareness: rho 0.032, p-value 0.818; Social Cognition: rho 0.11, p-value 0.442; Social Communication: rho 0.209, p-value 0.153; Social Motivation: rho 0.075, p-value 0.599; Autistic Mannerisms: 0.046, p-value 0.768; Total: rho -0.247, p-value 0.094) or the ADOS-2 comparison score (rho -0.241, p-value 0.169)

### 3.5 Results of comparison analysis (t-test for independent samples)

From the comparison analysis conducted on the basis of the presence/absence of ID, no statistically significant results were observed (t -1.146, p-value 0.094). Also, from the comparison analysis performed on the basis of sex (male/female), no statistically significant results were observed (t 0.164, p-value 0.871)

## 4 Discussion

### 4.1 Descriptive analysis

More male subjects were observed in our sample. This is in line with what is commonly reported in the literature regarding the epidemiology of ASD (28). The percentage of ID associated with ASD, on the other hand, is lower than in recent estimates (29), where it is around 30%. The prevalence of language impairment, on the other hand, is different from the recent literature: according to recent evidence, about 30% of individuals with ASD present as nonverbal or poorly verbal (30) while in our sample the prevalence of language impairment is about 54%. This difference could presumably be attributed to the difference in age of the sample members. Moreover, many patients were in the early stages of speech-language treatment at the time of assessment.

### 4.2 Correlation and regression analysis between FPI, SSP and ABC scales

From the comparison analysis performed between the FPI and sensory abnormalities, statistically significant correlations were observed between the variety of foods eaten and some sensory modalities, most of all with the scales “Taste/Smell Sensitivity” and “Tactile Sensitivity”, this according to the recent evidence (8). In fact, these sensory modalities are involved when eating; therefore, an abnormality of these modalities could justify unexpected or abnormal reactions for certain types of food. Both of these variables also were found to be directly and positively correlated with FPI scores: therefore, based on both questionnaires’ scoring, lower scores obtained on the SSP (i.e. greater sensory anomalies) correspond to greater FS (i.e. fewer food items accepted, as assessed by the FPI scale). Moreover, statistical significance was also observed for both of these modalities on regression analysis; therefore, we can conclude that sensory abnormalities in taste, smell and touch may predict worse FS. Of these analyses, we can observe how the B coefficient (which describes the relationship between the two variables) is positive for both, indicating a direct relationship between the dependent and independent variables. Of these, examining the value of R^2^, we can observe that for all analyses this value is low: therefore, despite statistical significance, sensory anomalies do not fully explain the presence of FS. At preliminary analysis, the “Hyporesponsiveness/sensory seeking” scale resulted as a predictor of FPI value. This result wasn’t expected, since there is no apparent association between FSy and sensory seeking behaviours. When the regression analysis was repeated and weighted for IQ level, no significant association was found, confirming the confounding effect of IQ levels in individuals with sensory seeking behaviours. Therefore, the predictive role of this scale on the FS y of subjects ASD was excluded. The predictive role of the scales “Auditory filtering” and “Low energy/weakness” was also excluded because the regression model was not found to be reliable. Therefore, what was observed in bivariate correlation analysis could represent incidental findings with no real impact on the clinical phenotype of subjects ASD. Overall, the association shown between sensory profile abnormalities and FSare in line with previous clinical studies that have shown similar results (31, 32). Regarding the correlation analysis between the FPI and the ABC scales, no statistically significant results were observed. The ABC scale investigates dysfunctional behaviors without any items that are directly related to any dysfunctional eating behavior. This could explain the different results compared with a similar study: *Wenzell M.L.* et al. (15), that found out a significant correlation between the “Hyperactivity, Lack of Compliance” scale and the diet of individuals with ASD, assessed using the *Brief Autism Mealtime Behavior Inventory* scale. However, this scale (33) evaluates possible dysfunctional behaviors present during mealtime, which could explain the results that emerged. Our results underline, instead, no significant correlations with other behavioral challenges, indicating FSonly related to sensory abnormalities.

### 4.3 Correlation analysis with autistic symptomatology

The analyses conducted showed no significant correlation between autistic symptomatology scores and FS in our sample. This finding wasn’t expected from the preliminary hypotheses: it is well known that repetitive and ritualistic behaviors are associated with greater FS (34). This result seems to support biologically determined basis of FSy (possible sensorial basis) rather than the behavioral one. It also excludes that the severity of autism-related symptoms can directly impact on eating style. These findings suggest also that FSshould be considered as a possible comorbidity of an eating disorder in ASD, such as avoidant/restrictive food intake disorder, rather than a symptom of ASD itself.

### 4.4 Comparison analysis

Comparison analysis (independent t-test) showed no statistically significant differences by dividing the sample into males/females, with/without ID, compared for the FPI variable. Considering two separate groups, males and females, there is current and expanding evidence of differences in developmental and functional profiles between males and females ASD, not only in the intensity of social and repetitive symptoms but also in the sensory profile as well as in the neurobiological organisation of the Central Nervous System (35, 36). To date, it seems that no previous study analysed FS comparing male and female subjects. Given the importance of the differential aspects of the clinical profile within ASD, further studies that can better characterise the FS based on sex are considered useful. The differences found in the dietary preferences between children with and without ID, on the other hand, appears difficult to interpret. A previous study assessed IQ differences based on the presence or absence of FSy and found significant results, with higher IQs in children without FS (15). In our sample, we performed a different analysis, dividing children based on cognitive level (with/without ID) and then comparing the two groups, considering the FPI values. This could explain why our results are different from the ones described by *Wenzell* et al. (15). Furthermore, the cognitive assessment was performed using several different instruments, considering the cooperation and developmental profile of the single child. This may have affected an unequivocal definition of ID.

### 4.5 Food selectivity and Autism Spectrum Disorder

FS is a very common behaviour in ASD subjects. Overall, it is recognised that there is a strong association between FS and sensory abnormalities, especially those related to the texture and taste of food, which may be 20 times more frequent in ASD subjects (37). FS can also occur in ASD subjects at different levels of severity. Specifically, in the case of strong FS there is sometimes a diagnosis of comorbidity with Avoidant/Restrictive Feeding Disorder (ARFID). This disorder is characterised by difficulties in achieving the correct nutritional intake and the need to resort to additional means such as artificial nutrition (1). Recent prevalence estimates report that 11.41% of ASD subjects may also present with a diagnosis of ARFID; this percentage would. therefore, be high enough to justify screening for ARFID in the case of ASD (38). The impact of FS is therefore not only in terms of rehabilitation management but also in terms of possible gastrointestinal disorders: nutritional imbalance can lead to inadequate intake of fibre, protein and essential fatty acids. Such aspects lead to the development of gastrointestinal disorders as well as an alteration of the intestinal microbiota (39). In ASD subjects, the most commonly reported gastrointestinal symptoms are constipation, diarrhoea and abdominal pain and their frequency is directly related to FS (40). In light of the above, it can be seen that individual rehabilitation programmes aimed at improving FS should be set up. For example, behavioural intervention programmes (including gradual exposure and positive reinforcement strategies) appear to be promising in the treatment of FS (41). In the context of such programmes, it also appears necessary to constantly involve parental figures, in order to maximise the results both in terms of food consumed and dysfunctional mealtime behaviour (42). Also in the context of FS, our study suggests a key role of sensory abnormalities in determining this behaviour. In the clinical setting, therefore, screening for sensory abnormalities could be useful for all subjects with ASD and FS. In addition, individuals with ASD could be initiated into specific rehabilitation programmes aimed at improving sensory aspects such as the Ayres Sensory Integration intervention, which has shown benefits in improving sensory integration of ASD subjects (43).

### 4.6 Strengths and limitations

The present study primarily aims to provide a phenotypic characterisation of a cohort of subjects ASD, with specific focus on how FSmay impact on their overall psychopathological profile. In the scientific literature, but also in clinical practice, assessment tools, based on parental and caregiver reports, are fundamental in order to determine behaviours that cannot be directly and daily observed, such as eating. The use of indirect tools, on the other hand, also represents a critical issue, because the report provided by the parents might not be accurate, while an integrated evaluation might represent a more accurate assessment. Another limitation of the study is also represented by the small sample size, which could have affected the inferential possibilities of the analyses conducted. In addition, the use of different instruments, rather than the ones presented by other studies, could explain the discordant results shown in literature. Specifically, the FPI is not frequently used as an assessment instrument, even if there are recent studies that aim to validate this tool (23). Finally, a limitation of the present study is its retrospective design. This aspect might have affected the completeness and quality of the collected data. The strength of this instrument is that it investigates all the possible foods consumed by the subject in relation to its consumption in the family. On the other hand, it does not explore possible dysfunctional behaviour present at mealtimes. Therefore, further studies could be useful to assess the actual usefulness of the FPI in the context of ASD, as well as expanding the study sample. An additional aspect concerning the present study could be the exclusion of subjects with concomitant psychiatric diagnoses such as ADHD. This exclusion criterion was adopted to avoid confounding factors in the interpretation of the results. In fact, the exclusion of comorbid psychiatric disorders was specifically performed to avoid the detection of abnormal scores on the ABC scale that could result from comorbid psychiatric conditions rather than ASD. However, to date, given the frequent comorbidity between the two disorders (44), the results we have reported may not be entirely consistent with what occurs in regular clinical practice.

## 5 Conclusion

In conclusion, our study has demonstrated a predictive role of sensory anomalies on FS. No significant associations were observed concerning behavioral profile, sex, IQ, or autistic symptoms. These results therefore suggest a key role of sensory anomalies in determining FS at different levels, as also indicated by previous literature. It is thus considered useful for all individuals with ASD to undergo screening for sensory anomalies, especially in the presence of FS, and for this aspect to be addressed in rehabilitative intervention. Furthermore, additional clinical studies are recommended to investigate the associations between FS and other elements of the clinical profile of individuals with ASD, preferably in larger cohorts monitored longitudinally over time

## Data Availability

The raw data supporting the conclusions of this article will be made available by the authors, without undue reservation.

## References

[1] American Psychiatric Association. Diagnostic and statistical manual of mental disorders (DSM-5^®^). Arlington, VA, USA: American Psychiatric Pub (2013).

[2] Shoaib A Cepeda MS Murray G Ochs-Ross R. Autism: comorbidities and treatment patterns in the real world, a retrospective cohort study among children, adolescents and adults newly diagnosed with autism. J Autism Dev Disord. (2022) 52:4311–20. doi: 10.1007/s10803-021-05289-x PMC9508210 34623581

[3] Muskens JB Velders FP Staal WG. Medical comorbidities in children and adolescents with autism spectrum disorders and attention deficit hyperactivity disorders: a systematic review. Eur Child Adolesc Psychiatry. (2017) 26:1093–103. doi: 10.1007/s00787-017-1020-0 PMC5591355 28674760

[4] Margari L Marzulli L Gabellone A de Giambattista C. Eating and mealtime behaviors in patients with autism spectrum disorder: current perspectives. Neuropsychiatr Dis Treat. (2020) 16:2083–102. doi: 10.2147/NDT.S224779 PMC7504729 32982247

[5] Molina-López J Leiva-García B Planells E Planells P. Food selectivity, nutritional inadequacies, and mealtime behavioral problems in children with autism spectrum disorder compared to neurotypical children. Int J Eat Disord. (2021) 54(12):2155–66. doi: 10.1002/eat.v54.12 34704615

[6] Sharp WG Postorino V McCracken CE Berry RC Criado KK Burrell TL. Dietary intake, nutrient status, and growth parameters in children with autism spectrum disorder and severe food selectivity: an electronic medical record review. J Acad Nutr Diet. (2018) 118(10):1943–50. doi: 10.1016/j.jand.2018.05.005 30005820

[7] Leader G O’Reilly M Gilroy SP Chen JL Ferrari C Mannion A. Comorbid feeding and gastrointestinal symptoms, challenging behavior, sensory issues, adaptive functioning and quality of life in children and adolescents with autism spectrum disorder. Dev Neurorehabil. (2021) 24:35–44. doi: 10.1080/17518423.2020.1770354 32496834

[8] Bandini LG Anderson SE Curtin C Cermak S Evans EW Scampini R. Food selectivity in children with autism spectrum disorders and typically developing children. J Pediatr. (2010) 157(2):259–64. doi: 10.1016/j.jpeds.2010.02.013 PMC2936505 20362301

[9] Hubbard KL Anderson SE Curtin C Must A Bandini LG. A comparison of food refusal related to characteristics of food in children with autism spectrum disorder and typically developing children. J AcadNutr Diet. (2014) 114:1981–7. doi: 10.1016/j.jand.2014.04.017 PMC4252256 24928779

[10] Chistol LT Bandini LG Must A Phillips S Cermak SA Curtin C. Sensory sensitivity and food selectivity in children with autism spectrum disorder. J Autism Dev Disord. (2018) 48:583–91. doi: 10.1007/s10803-017-3340-9 PMC6215327 29116421

[11] Gomes E Pedroso FS Wagner MB. Auditory hypersensitivity in the autistic spectrum disorder. Pró-Fono R Atual Cient (2008) 20(4):279–84. doi: 10.1590/s0104-56872008000400013 19142473

[12] Griffin ZAM Boulton KA Thapa R DeMayo MM Ambarchi Z Thomas E. Atypical sensory processing features in children with autism, and their relationships with maladaptive behaviors and caregiver strain (published online ahead of print, 2022 Mar 17). Autism Res. (2022) 15(6):1120–9. doi: 10.1002/aur.2700 PMC9544661 35297186

[13] Mendive Dubourdieu P Guerendiain M. Dietary intake, nutritional status and sensory profile in children with autism spectrum disorder and typical development. Nutrients. (2022) 14(10):2155. doi: 10.3390/nu14102155 35631297 PMC9147144

[14] Leader G Tuohy E Chen JL Mannion A Gilroy SP. Feeding problems, gastrointestinal symptoms, challenging behavior and sensory issues in children and adolescents with autism spectrum disorder. J Autism Dev Disord. (2020) 50:1401–10. doi: 10.1007/s10803-019-04357-7 31955310

[15] Wenzell ML Pulver SL McMahon MXH Rubio EK Gillespie S Berry RC. Clinical correlates and prevalence of food selectivity in children with autism spectrum disorder. J Pediatr. (2024) 269:114004. doi: 10.1016/j.jpeds.2024.114004 38447756

[16] Lord C Rutter M DiLavore P Risi S Gotham K Bishop S. Autism diagnostic observation schedule: ADOS-2. Western psychol Services. (2012).

[17] Bölte S Poustka F Constantino JN. Assessing autistic traits: cross-cultural validation of the social responsiveness scale (SRS). Autism Res. (2008) 1:354–63. doi: 10.1002/aur.49 19360690

[18] Lanfranchi S Rea M Vianello R Ferri R. Griffiths-III, italian edition. Florence, Italy: Hogrefe Editore (2016).

[19] Roid GH Miller LJ. Leiter international performance scale-revised (Leiter-R). Wood Dale, IL: Stoelting (1997).

[20] Wechsler Intelligence Scale for Children – Quarta Edizione WISC-IV. Manuale di somministrazione e scoring. Firenze: Giunti O.S. Organizzazioni Speciali (2012).

[21] Balboni G Belacchi C Bonichini S Coscarelli A. Vineland-II. Vineland adaptive behavior scales second edition-survey form-standardizzazione italiana. Firenze, Italia: Giunti O.S. (2016).

[22] Schreck KA Williams K. Food preferences and factors influencing food selectivity for children with autism spectrum disorders. Res Dev Disabil. (2006) 27(4):353–63. doi: 10.1016/j.ridd.2005.03.005 16043324

[23] Riccio MP Franco C Negri R Ferrentino RI Maresca R D'alterio E. Is food refusal in autistic children related to TAS2R38 genotype? Autism Res. (2018) 11(13):531–8. doi: 10.1002/aur.1912 29282878

[24] Aman MG Singh NN. Aberrant behavior checklist-community. In: Supplementary manual. Slosson Educational Publications, New York (1994).

[25] Kaat AJ Lecavalier L Aman MG. Validity of the aberrant behavior checklist in children with autism spectrum disorder. J Autism Dev Disord. (2014) 44:1103–16. doi: 10.1007/s10803-013-1970-0 24165702

[26] McIntosh DN Miller LJ Shyu V Dunn W. Overview of the short sensory profile. In: Dunn W, editor. Sensory Profile user’s manual. San Antonio: Pearsons (1999). pp. 59–73.

[27] Dunn W. Performance of typical children on the Sensory Profile: an item analysis. Am J OccupTher. (1994) 48:967–974. doi: 10.5014/ajot.48.11.967 7530904

[28] Becker KG. Male gender bias in autism and pediatric autoimmunity. Autism Res. (2012) 5:77–83. doi: 10.1002/aur.1227 22431266 PMC4530611

[29] Zeidan J Fombonne E Scorah J Ibrahim A Durkin MS Saxena S. Global prevalence of autism: A systematic review update. Autism Res. (2022) 15(5):778–90. doi: 10.1002/aur.2696 PMC9310578 35238171

[30] Brignell A Chenausky KV Song H Zhu J Suo C Morgan AT. Communication interventions for autism spectrum disorder in minimally verbal children. Cochrane Database Syst Rev. (2018) 11:CD012324. doi: 10.1002/14651858.CD012324.pub2 30395694 PMC6516977

[31] Nadon G Feldman DE Dunn W Gisel E. Association of sensory processing and eating problems in children with autism spectrum disorders. Autism Res Treat. (2011) 2011:541926. doi: 10.1155/2011/541926 22937249 PMC3420765

[32] Panerai S Ferri R Catania V Zingale M Ruccella D Gelardi D. Sensory profiles of children with autism spectrum disorder with and without feeding problems: A comparative study in sicilian subjects. Brain Sci. (2020) 10(6):336. doi: 10.3390/brainsci10060336 32486513 PMC7349225

[33] DeMand A Johnson C Foldes E. Psychometric properties of the brief autism mealtime behaviors inventory. J Autism Dev Disord. (2015) 45:2667–73. doi: 10.1007/s10803-015-2435-4 PMC4554795 25813517

[34] Byrska A Błażejczyk I Faruga A Potaczek M Wilczyński KM Janas-Kozik M. Relationships between feeding problems, behavioral characteristics and nutritional quality in children with ASD. Patterns of food selectivity among children with autism spectrum disorder. J Clin Med. (2023) 12:5469. doi: 10.3390/jcm12175469 37685537 PMC10488249

[35] Napolitano A Schiavi S La Rosa P Rossi-Espagnet MC Petrillo S. Sex differences in autism spectrum disorder: diagnostic, neurobiological, and behavioral features. Front Psychiatry. (2022) 13:889636. doi: 10.3389/fpsyt.2022.889636 35633791 PMC9136002

[36] Lai MC Lombardo MV Auyeung B Chakrabarti B Baron-Cohen S. Sex/gender differences and autism: setting the scene for future research. J Am Acad Child Adolesc Psychiatry. (2015) 54:11–24. doi: 10.1016/j.jaac.2014.10.003 25524786 PMC4284309

[37] Mørdre M Ørbeck B Hoel RE Øvergaard KR. Food selectivity in children and adolescents with autism spectrum disorders - a systematic literature review. Selektive spisemønstre hos barn og unge med autismespekterforstyrrelser – en systematisk litteraturoversikt. Tidsskrift den Norske laegeforening: tidsskrift praktisk medicin ny raekke. (2024) 144. doi: 10.4045/tidsskr.24.0193 39605156

[38] Sader M Weston A Buchan K Kerr-Gaffney J Gillespie-Smith K Sharpe H. The co-occurrence of autism and avoidant/restrictive food intake disorder (ARFID): A prevalence-based meta-analysis. Int J eating Disord. (2025) 58:473–88. doi: 10.1002/eat.24369 PMC11891632 39760303

[39] Valenzuela-Zamora AF Ramírez-Valenzuela DG Ramos-Jiménez A. Food selectivity and its implications associated with gastrointestinal disorders in children with autism spectrum disorders. Nutrients. (2022) 14:2660. doi: 10.3390/nu14132660 35807840 PMC9268444

[40] Babinska K Celusakova H Belica I Szapuova Z Waczulikova I Nemcsicsova D. Gastrointestinal symptoms and feeding problems and their associations with dietary interventions, food supplement use, and behavioral characteristics in a sample of children and adolescents with autism spectrum disorders. Int J Environ Res Public Health. (2020) 17:6372. doi: 10.3390/ijerph17176372 32882981 PMC7503400

[41] Al-Beltagi M. Nutritional management and autism spectrum disorder: A systematic review. World J Clin Pediatr. (2024) 13:99649. doi: 10.5409/wjcp.v13.i4.99649 39654662 PMC11572612

[42] Burrell TL Scahill L Nuhu N Gillespie S Sharp W. Exploration of treatment response in parent training for children with autism spectrum disorder and moderate food selectivity. J Autism Dev Disord. (2023) 53:229–35. doi: 10.1007/s10803-021-05406-w 35032300

[43] Schoen SA Lane SJ Mailloux Z May-Benson T Parham LD Smith Roley S. A systematic review of ayres sensory integration intervention for children with autism. Autism research: Off J Int Soc Autism Res. (2019) 12:6–19. doi: 10.1002/aur.2046 PMC6590432 30548827

[44] Mutluer T Aslan Genç H Özcan Morey A Yapici Eser H Ertinmaz B Can M. Population-based psychiatric comorbidity in children and adolescents with autism spectrum disorder: A meta-analysis. Front Psychiatry. (2022) 13:856208. doi: 10.3389/fpsyt.2022.856208 35693977 PMC9186340

